# A protocol for systematic review and meta-analysis of optimizing treatment for malaria

**DOI:** 10.1097/MD.0000000000022044

**Published:** 2020-09-04

**Authors:** Renyan Zhang, Xing Dong, Junyao Wang, Ying Guo, Yong Dai

**Affiliations:** School of Basic Medicine, Chengdu University of Traditional Chinese Medicine, Chengdu, China.

**Keywords:** artemisinin combination therapies, malaria, meta-analysis, protocol, systematic review

## Abstract

**Background:**

Malaria remains a global health threat for centuries. In recent years, a rising resistance of Plasmodium falciparum to current standard artemisinin-based combination therapies (ACTs) leads to increasing treatment failures and requires for optimized treatment. Here, we intend to make a systematic review and meta-analysis of optimizing treatment for malaria, so as to find a potential optimal treatment.

**Methods:**

We will search electronic databases: the Cochrane Infectious Diseases Group (CIDG) Specialized Register, the Cochrane Central Register of Controlled Trials (CEN-TRAL), PubMed, Embase, Web of Science from their inception to 1 July, 2020. We will also search International Clinical Trials Registry Platform (ICTRP) and ClinicalTrials.gov, and contact with authors when necessary. Two authors will independently collect and select data, and the statistical analyses will be conducted by Revman V.5.3 software.

**Results:**

We will evaluate efficacy and safety of modified ACTs for uncomplicate malaria, comparing with standard ACTs in all eligible clinical studies.

**Conclusion:**

In this study, we will offer clinical evidence for optimizing treatment for malaria.

**Registration number:**

INPLASY202070115

## 1 Introduction

Malaria is a mosquito-borne parasitic disease, causing 400 thousand deaths or so globally every year, with most dead cases in Africa and South Asia.^[1]^ There are 6 species of plasmodium can cause human malaria, namely *Plasmodium falciparum*, *Plasmodium vivax*, *Plasmodium malariae, Plasmodium ovale curtisi, Plasmodium ovale wallikeri* and *Plasmodium knowlesi*,^[2]^ among which *P falciparum* claims for the majority of morbidity and mortality, and is much prone to develop resistance to current antimalarial agents. The malaria infection can result in a spectrum of clinical symptoms, characterized by fever, chills, anemia, headache, nausea, muscle pain and severe malaria (including cerebral malaria, severe anemia, acute respiratory stress, multiple organ failure) and even death.^[3,4]^ After the discovery and utility of quinine extracted from bark of the cinchona tree, the morbidity and fatality number had sharply declined, which lost efficacy not long after using owing to resistance, unfortunately. The malaria cases of morbidity and fatality had gone thought a boost for a long period until a Chinese scientist Yoyo Tu extracted artemisinin from leaves of *Artemisia Annua Linn.*, which was inspired by *Zhou Hou Bei Ji Fang*, one of ancient Chinese Medicine Classics, that serves as a handbook of prescriptions for emergencies.^[5]^

In light of the occurrence of resistance to quinine, World Health Organization (WHO) has recommended artemisinin-based combination therapies (ACTs) as standard treatment for uncomplicated malaria since 2005.^[6]^ Nevertheless, an increasing number of treatment failures due to resistance found in Southeast Asia, manifesting as delayed parasite clearance time, worries people of global malaria control and elimination.^[7–10]^ Researchers have been working on developing effective vaccines for malaria which has been well reviewed.^[11]^ There are several vaccines are undergoing clinical trial phases,^[12–16]^ among which *RTS,S* was reported to be successful in preventing nearly 30% onset of severe malaria, and is now under phase 3 clinical trial.^[17]^ Without available vaccine and novel antimalarial drugs yet, ACTs are still top choice among all therapies for now, though there are thousands of candidate chemicals and hundreds of compounds showing promising antimalarial effects in preclinical stage and clinical trials.^[18,19]^ To make the maximum use of ACTs, proper alterations in standard ACTs could be benefit for containing malaria cases all over the world. In this study, we will evaluate the safety and efficacy of other interventions in RCTs of included studies, so as to provide evidence for modifications of standard ACTs to sustain the progress we have made in malaria control over the years.

## 2 Methods

### 2.1 Study registration

This meta-analysis was registered with the International Platform of Registered Systematic Review and Meta-Analysis Protocols (INPLASY) on 26 July, 2020 (registration number INPLASY202070115). The Preferred Reporting Items for Systematic Review and Meta-Analysis Protocols (PRISMA-P) 2015 statement^[20]^ and the Cochrane Handbook for Systematic Reviews of Interventions^[21]^ (CHSRI) will be carefully followed throughout the process.

### 2.2 The inclusion criteria

#### 2.2.1 Types of studies

Studies that report randomized controlled trials (RCTs) and quasi-RCTs of comparison between standard ACTs and other administrations for uncomplicated malaria are considered for inclusion in this study. Studies involving non-RCTs, animal experiments, case reports, reviews will be excluded.

#### 2.2.2 Types of participants

Children over 5 years of age and unpregnant adults who are diagnosed with uncomplicated malaria regardless of malaria species are eligible for our study. Patients treated with antimalarial agents within 48 hours before enrollment or allergic to any drug of ACT will be excluded. The nationality, race, sex, educational or financial status, and occupation are not limited. Patients diagnosed with severe malaria will not be included.

#### 2.2.3 Types of interventions

The interventions of the experimental group in this study include ACTs with either additional antimalarial agents or modified combination of the standard ACTs in both types of medicine, dosage and course.

#### 2.2.4 Types of outcomes

The main outcomes are parasitemia in patient's peripheral blood smears and parasite clearance time. The secondary outcomes are body temperature (°C), weight (kg), hemoglobin (g/dl), red blood cell (RBC) count(×10^12^/l), white blood cell (WBC) count (×10^9^/l), platelet count (×10^9^/l), Alanine aminotransferase (ALT) (U/l) and Aspartate aminotransferase (AST) (U/l).

### 2.3 Collection and analysis of data

#### 2.3.1 Search strategy

Author Wang will carry out a thorough search in electronic databases: the Cochrane Infectious Diseases Group (CIDG) Specialized Register, the Cochrane Central Register of Controlled Trials (CEN-TRAL), PubMed, Embase, Web of Science from their inception to 1 July, 2020. We will also search the International Clinical Trials Registry Platform (ICTRP) and ClinicalTrials.gov, using medical subject headings from Mesh plus free words to get complete searching results. We will contact with authors when necessary. The detailed information of electronic search is listed in Table 1.

**Table 1:**
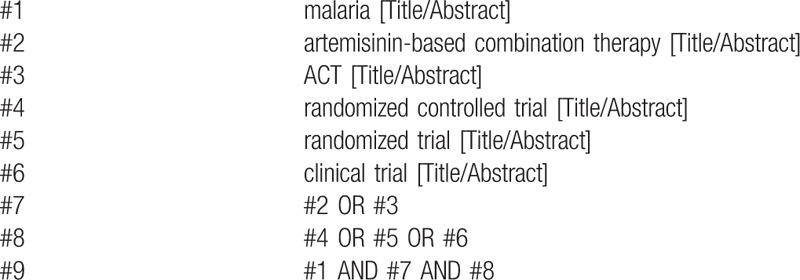
Search strategy performed in PubMed.

#### 2.3.2 Selection and extraction of data

**Figure 1:**
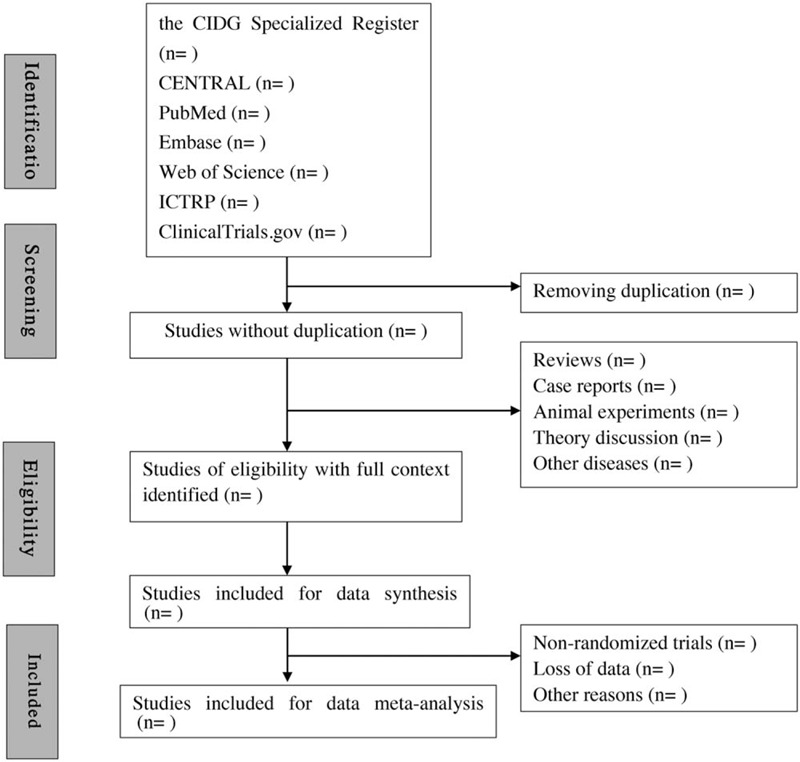
Flow diagram of studies selection process.

All of the authors will be trained with PRISMA-P and CHSRI from the beginning. Zhang and Wang will independently select data and check the title, abstract, and content of all enrolled studies for their eligibility. All repetitions and studies not met the inclusion criteria will be excluded. Then, they will cross-check their included and excluded studies. Study with uncertainty will be presented to the third author or the panel to judge if they are eligible for this study. The standard data extraction table will be determined before data extraction. Author Zhang and Wang will independently extract data in terms of general information, method description, participant and intervention, outcomes and measures and annotations of selected data. When there is uncertain study, we will have expert discussion to make a final decision. When necessary, we will contact with the authors for more related information and clinical data. Studies with no access to the original paper will be excluded. When study contains multiple groups, we will only extract data meet the inclusion criteria of this study. The diagram of this study is shown in Figure 1.

#### 2.3.3 Assessment of risk of bias and quality of evidence

The two authors will independently evaluate the methodology quality with the Cochrane's risk of bias tool, Review Manager 5.3 (Revman V.5.3), basing on the CHSRI. All uncertain studies will be presented to the panel or consult with experts. The quality of evidence will be assessed in the guidance of the Grading of Recommendations, Assessment, Development, and Evaluation (GRADE) method. The following aspects will be involved: random sequencing generation, allocation concealment, blinding of outcomes, completeness of outcomes and measures, reporting bias and other sources of biases, for instance, conflicts of interest etc.

### 2.4 Statistical analysis

#### 2.4.1 Synthesis of data

Basing on the principle of participants, interventions, comparators, and outcomes (PICO), we will draw a basic feature table of all included studies that indicates all the general information and differences in methodology, and help us determine whether the clinical data can be quantitatively synthesized.

#### 2.4.2 Measures of effect size

Author Dong will perform the statistical analyses with Revman V.5.3 software. The dichotomous data will be processed with risk ratio (RR) and a 95% confidence interval (CI) will be calculated. The weighted mean difference (WMD) or standard mean difference (SMD) with a 95% CI will be calculated as for continuous outcomes.

#### 2.4.3 Assessment of heterogeneity

The heterogeneity of included data will be assessed by the Cochrane's Q test and be quantified with *I*^*2*^ test. We will adopt a fixed effect model with *I*^*2*^ > 50%, while a random effect model with *I*^*2*^ ≤ 50% will be declined.

#### 2.4.4 Sensitivity analysis

We will evaluate the stability of all included results by excluding the homogeneity. When studies are of low quality, we will perform a sensitivity analysis with Revman V.5.3.

#### 2.4.5 Assessment of reporting bias

Reporting bias will be evaluated when more than 10 studies are included by making funnel plots.

#### 2.4.6 Subgroup analysis

When necessary, we will conduct a subgroup analysis of the included studies with high heterogeneity, in order to find out source of heterogeneity, which may also lead us to a certain population that are more sensitive to the regimen.

## 3 Discussion

Malaria is still a leading killer of hundreds of thousands of lives globally, of which children under 5 years of age and pregnant women account for the majority. With the discovery and development of quinine and artemisinin, we have achieved great progress in malaria control. In order to delay the occurrence of resistance to artemisinin drugs, WHO has recommended ACTs as the standard treatment of uncomplicated malaria since early 2000s. However, a long-term abuse of nonstandard monotherapy in treating malaria in certain areas has induced multiple plasmodium, especially of falciparum species, to develop resistance to almost any of current frontline antimalarial agents, which will vastly challenge the progress we have made over the years.^[22]^ People now are concerned that the increasing number of treatment failures in South Asia may quickly spread globally, especially Africa where burned the majority of mortality all over the world. Though there are many vaccines undergoing clinical trials for years, no vaccine has been proven to be highly safe and effective so far. Without effective vaccine, we can only depend on medication to treat malaria. In this study, we will review all related clinical reports of new modification on the standard ACTs and analyze the clinical data to evaluate the safety and effectiveness of these therapies and to provide solid evidence for potential alternative ACT treatment in future utility. We will try to thoroughly search the electronic databases, clinical online registration websites and get in touch with authors of included studies for more detailed information to get a complete data as possible. But we have to admit that there could be limitations for possible lack of related unpublished documents, and the scale or quality of RCTs that we include may affect our analyses. We hope that more multiple-center RCTs of large scale and of high quality could be reported to offer more solid evidence in the future.

## Author contributions

**Conceptualization:** Renyan Zhang

**Data collection:** Junyao Wang, Renyan Zhang

**Software:** Xing Dong

**Statistical analysis:** Xing Dong

**Supervision:** Ying Guo

**Writing – original draft:** Renyan Zhang

**Writing – review & editing:** Yong Dai
